# Identification of very small open reading frames in the genomes of Holmes Jungle virus, Ord River virus, and Wongabel virus of the genus *Hapavirus*, family *Rhabdoviridae*

**DOI:** 10.1177/1176934317713484

**Published:** 2017-07-11

**Authors:** Aneta Gubala, Susan Walsh, Jane McAllister, Richard Weir, Steven Davis, Lorna Melville, Ian Mitchell, Dieter Bulach, Penny Gauci, Alex Skvortsov, David Boyle

**Affiliations:** 1 Land Division, Defence Science and Technology Group, Fishermans Bend, VIC, Australia; 2 Berrimah Veterinary Laboratories, Department of Primary Industry and Fisheries, Northern Territory Government, Berrimah, NT, Australia; 3 Australian Animal Health Laboratory, Commonwealth Scientific and Industrial Research Organisation, Geelong, VIC, Australia

**Keywords:** Holmes Jungle virus, rhabdovirus, genome sequence, smORFs, *Culex annulirostris*

## Abstract

Viruses of the family *Rhabdoviridae* infect a broad range of hosts from a variety of ecological and geographical niches, including vertebrates, arthropods, and plants. The arthropod-transmitted members of this family display considerable genetic diversity and remarkable genomic flexibility that enable coding for various accessory proteins in different locations of the genome. Here, we describe the genome of Holmes Jungle virus, isolated from *Culex annulirostris* mosquitoes collected in northern Australia, and make detailed comparisons with the closely related Ord River and Wongabel viruses, with a focus on identifying very small open reading frames (smORFs) in their genomes. This is the first systematic prediction of smORFs in rhabdoviruses, emphasising the intricacy of the rhabdovirus genome and the knowledge gaps. We speculate that these smORFs may be of importance to the life cycle of the virus in the arthropod vector.

## Introduction

The family *Rhabdoviridae* of the order *Mononegavirales* is one of the largest and most diverse virus families described to date.^1^ These negative-sense single-stranded RNA (−ssRNA) viruses are known to infect a broad range of hosts, including plants, arthropods, birds, fish, reptiles and mammals. In recent times, numerous novel or poorly understood rhabdoviruses from ecologically and geographically distinct regions have undergone detailed genetic characterisation. A unique and important group of insect-borne rhabdoviruses, known as the Hart Park group (recently ratified by the International Committee on Taxonomy of Viruses as a new genus *Hapavirus*),^2^ has emerged and progressively expanded to include many members being subject to comprehensive genetic and evolutionary investigations.^3–7^ Many viruses of this group have a proclivity for mosquito and bird hosts, and the founding members of this group, Hart Park virus (HPV, isolated in 1951) and Flanders virus (FLAV, isolated in 1961), are known to circulate between culicine mosquitoes and passerine birds in Western and Eastern United States, respectively.^3,8–11^ The presence of related viruses in Australia was identified following the genomic characterisation of the bird-associated Wongabel virus (WONV), isolated from *Culicoides austropalpalis*, biting midges from the Atherton Tablelands in northern Queensland.^6,12,13^ Subsequent to this, the genomic characterisation of another related Australian virus, Ngaingan virus (NGAV, isolated from *Culicoides brevitarsis*/*actoni* collected from the Gulf of Carpentaria, northern Queensland) revealed an atypical host range (kangaroos, wallaroos, wallabies and livestock) and a remarkably large and complex genome structure not previously observed in rhabdoviruses.^5,14–16^

Antigenic cross-reactivity studies of rhabdoviruses in the 1980s led to the suggestion that the Hart Park group should constitute a new genus.^17^ The identification of WONV and NGAV in Australia further supported this view. Subsequent microarray and genomic sequencing studies identified numerous additional members to this group and led to the proposal of a new genus *Hapavirus*.^2,7,18^ The recently characterised viruses that are now members of this genus include Kamese virus (KAMV), Mossuril virus (MOSV) and Mosqueiro virus (MQOV), which were previously known to antigenically cross-react with HPV.^17–23^ These viruses were isolated from various *Culex* mosquitoes from Uganda, Mozambique, and Brazil, respectively. Serological evidence suggests that KAMV and MOSV can infect humans and MOSV antibodies have also been found in a baboon. Marco virus (MCOV) isolated from the lizard *Ameiva ameiva ameiva* from Brazil previously tested negative for antigenic cross-reactivity with other hapaviruses^17^; however, it too is now a member of the genus.^7,24^ Other recently identified members of this genus include Gray Lodge virus (GLOV; USA), Joinjakaka virus (JOIV; Papua New Guinea), La Joya virus (LJV; Panama) and Manitoba virus (MANV; Canada), all isolated from culicine mosquitoes, and Landjia virus (LJAV; Central African Republic) isolated from passerine birds. Collectively, these newly characterised viruses have extended the known host, vector and ecological ranges of this group.

Holmes Jungle virus (HOJV) and Ord River virus (ORV) were isolated in Australia from the *Culex annulirostris* mosquito, which is an important vector for several arboviruses of public health significance in Australia including Ross River, Murray Valley encephalitis, Barmah Forest, Kunjin and Japanese encephalitis. HOJV was isolated near Darwin, Northern Territory, Australia in 1987.^25^ Previously described serological surveys found antibodies to HOJV in cattle in the Northern Territory (9/2250; 0.4%) and Cape York (3/219; 1.4%), and HOJV, or a closely related virus, has also been detected in Indonesian cattle.^25^ Antibodies to HOJV have also been detected in a hospitalised patient, although there was no definitive link with disease (Weir, unpublished data). ORV was isolated from the Kununurra region in Western Australia during investigations performed by the University of Western Australia into the distribution of arboviruses in the north of the state between the years 1972 and 1976.^26,27^ The vertebrate hosts of ORV are not known. Complete genome sequencing and phylogenetic analysis of HOJV and ORV described here, and the recently published near-complete genome sequence of ORV,^7^ demonstrate that these viruses cluster with WONV within the genus *Hapavirus*.

Rhabdoviruses contain a −ssRNA genome which encodes five structural proteins: the nucleoprotein (N), phosphoprotein (P), matrix protein (M), glycoprotein (G), and the polymerase (L). The N protein of rhabdoviruses is associated with the viral genomic RNA to form the ribonucleocapsid complex that forms the core of the virion and acts as template for transcription and replication by the L polymerase and the P phosphoprotein.^28–30^ The M protein has a diverse range of roles including assembly of the virion by interacting with the ribonucleocapsid, lipid membranes and the cytoplasmic tails of the G protein, and mediating virus budding from the cell.^31–33^ The M protein also has a role in blocking host anti-viral gene products.^34–36^ The G protein of rhabdoviruses forms trimers that project outside of the surface of the virion; it is responsible for attachment to host cell receptors, membrane fusion, and is the target of host neutralising antibodies.^37^

In addition to the five structural proteins, the presence of accessory open reading frames (ORFs) is now recognised in several groups of animal rhabdoviruses. These are assumed to have an auxiliary role during infection and are not part of the virion structure. Accessory ORFs were first observed in rhabdoviruses of plants and lower vertebrates (fish),^38,39^ followed by arthropod-transmitted viruses of bovines from the genus *Ephemerovirus*.^40–43^ Ephemeroviruses contain several accessory ORFs between the G and L ORFs (referred to as α, β, γ, and δ) and a second glycoprotein ORF (G_NS_). Additional ORFs were subsequently described in other higher-vertebrate arthropod-borne rhabdoviruses including WONV, NGAV and viruses of the genus *Tibrovirus* (Tibrogargan virus, TIBV; Coastal Plains virus, CPV; and Bas-Congo virus, BASV), taking on the naming convention of ‘U’ for ‘Unknown’ function.^5,6,44,45^ In recent times, many other previously uncharacterised rhabdoviruses were revealed to contain a variety of accessory ORFs.^7^ In general, accessory ORFs from different groups or genera do not appear to share recognisable nucleotide or amino acid sequence identity; therefore, predictive comparisons are difficult to make. However, some of the ORFs have been putatively grouped into categories based on similar predicted structures and characteristics.^7^ These categories include viroporins, small transmembrane proteins, small hydrophobic proteins, large class I transmembrane glycoproteins and other genus-specific accessory proteins.

Of all the different groups of viruses in the family *Rhabdoviridae*, the genus *Hapavirus* appears to contain the highest variability in genetic content between members of the group. Although most hapaviruses contain three accessory ORFs located between the P and M genes and one between the G and L genes, there are several members of this genus that contain discernibly different genome structures. For example, NGAV contains at least four additional ORFs within the G-L gene junction including a second glycoprotein gene (G_NS_) that is comparable with that found in the genus *Ephemerovirus*, which to date has served as a taxonomic marker for assigning viruses into this genus.^1,5^ Conversely, MCOV does not contain any ORFs between P and M, but it does contain two ORFs between G and L.^7^ The reasons and processes responsible for such large genetic disparities within a group of closely related rhabdoviruses are not yet well-understood; however, there appears to be a common process, most likely duplication of the upstream genes, which is responsible for the creation of new genes in rhabdoviruses.^7^ It is evident, with hapaviruses as examples, that rhabdovirus genomes are highly flexible in their ability to code for additional proteins, resulting in marked genome expansion or contraction with apparent minimal detriment to the replication and success of the virus.^7^

Most of the accessory ORFs found in hapaviruses to date are relatively large and located discernibly between the structural protein genes.^3,5–7^ They often contain independent transcription control signals indicating that they are autonomously transcribed and translated in the canonical manner that is traditionally observed in −ssRNA viruses. There is, however, sufficient evidence to indicate that some viruses use unconventional polycistronic transcription and translation mechanisms that enable non-canonical gene expression.^7,46^ There are numerous instances where the accessory ORFs overlap other genes in some rhabdoviruses, including in hapaviruses, indicating that these ORFs can only be transcribed on polycistronic messenger RNAs (mRNAs). This may be a particularly useful mechanism for controlling transcription and translation of accessory ORFs by the virus. This apparent capability raises the possibility for the translation of proteins from other very smORFs present within the genome.

There has been extensive study of smORFs in other organisms, including bacteria, plants, yeast, worms, flies and humans, as previously reviewed by Chu et al.^47^ For example, two smORF products (28 and 29 amino acids [aa] in length) found in drosophila (fruit fly) genomes were recently identified to be important in regulation of calcium transport and intake into drosophila muscle and heart cells.^48^ Analogues were subsequently identified in other species including humans and were suggested to have a fundamental role in cardiac and skeletal muscle calcium regulation.^49–51^ Several viruses of plants, insects and fungi contain smORFs that are viral suppressors of RNA silencing.^52–54^ We thus suggest that smORFs are probably abundant in many rhabdovirus genomes but are typically undetected due to their small size and uncertain significance. Therefore, in addition to the analysis of the expected ORFs, this report describes the first systematic in-depth analysis of smORFs predicted in three closely related rhabdoviruses (HOJV, ORV, and WONV of the genus *Hapavirus)*. Although the significance of smORFs in rhabdovirus genomes is purely speculative, their conservation and the similar features identified in all three viruses are suggestive of functional roles and indicate that this is an area that merits further study.

## Materials and Methods

### Virus propagation and RNA extraction

HOJV (strain DPP1163) and ORV (strain OR1023) were isolated from *C. annulirostris* mosquitoes. HOJV was isolated from Darwin, Northern Territory, Australia in 1987,^25^ and ORV was isolated from the Kununurra region, Western Australia in 1976 (unpublished data) during virus distribution studies in North Western Australia.^26,27^ Viruses were propagated in baby hamster kidney BHK-BSR cells (a derivative of the BHK-21 cell line); the infected tissue culture supernatant was collected and clarified at 4 to 5 days after infection at the first signs of cytopathic effect (CPE), as previously described, and ultracentrifuged at 70 000 × g for 1 hour in a Beckman 70Ti Rotor (Beckman Coulter, Sydney, Australia).^5^ RNA was extracted, as previously described, and subsequently used downstream for sequencing.^6^

### Sequencing

HOJV was sequenced using the previously described polymerase chain reaction (PCR)–select complementary DNA (cDNA) subtraction and traditional Sanger sequencing method.^6^ ORV RNA was reverse transcribed to double-stranded cDNA using the SuperScript Double-Stranded cDNA Synthesis Kit and 100 pmol random hexamer, as per the manufacturer’s instructions (Invitrogen, USA), and sequenced using next-generation sequencing on the Illumina GAIIx analyser by the Micromon service facility at Monash University (Melbourne, VIC, Australia) using procedures previously described.^55^ Where required, sequences were confirmed using Sanger sequencing, and the genome termini were sequenced using a modified rapid amplification of cDNA ends (RACE) method.^6^ Sequence data for both viruses were managed using programs as previously described,^5^ with the exception that ORV high-throughput sequence data were assembled and managed using Velvet 1.1.04, Geneious Pro 5.4, and Artemis (Sanger), as previously described.^56^

### Predictive sequence analyses

Sequence similarity searches of GenBank/EMBL and Swiss-Prot databases were performed using BLAST (Basic Local Alignment Search Tool) (http://www.ncbi.nlm.nih.gov). Pairwise (local and global) and multiple (ClustalW and MUSCLE) sequence alignments were performed using tools available at the EMBL-EBI server (https://www.ebi.ac.uk/) and at the CBS prediction server (www.cbs.dtu.dk). A similarity plot of HOJV and ORV genome sequences versus the WONV genome sequence was created using ClustalW-aligned sequences and SimPlot (v 3.5.1).^57^ Protein sequences were analysed using PredictProtein (http://www.predictprotein.org), ProtScale and SignalP programs (http://au.expasy.org), and Signal-BLAST (https://www.came.sbg.ac.at/). Transmembrane predictions were made using several programs: PHDhtm algorithm (neural networks) using PredictProtein; TMHMM Server 2 (hidden Markov model) at the www.cbs.dtu.dk interface; Phobius (homology-supported prediction including signal peptide prediction) available at http://phobius.sbc.su.se/; and HMMTOP v 2.0 available at http://www.enzim.hu/hmmtop/index.php.

To identify whether any of the predicted smORF protein products from HOJV, ORV, and WONV were similar in sequence, batched global pairwise alignments (BLOSUM50, gap opening penalty 10, and extension penalty 2) were performed sequentially with each of the proteins, using the University of Virginia FASTA server (http://fasta.bioch.virginia.edu/fasta_www2/fasta_www.cgi). Subsequently, the identified matching pairs of proteins were visualised using the global EMBOSS Needle pairwise alignment tool (EBLOSUM62, gap opening 10, gap extension 0.5) at the www.cbs.dtu.dk interface for depiction in Figures 5 and 6. Due to the extremely small sizes of the protein products of the smORFs, BLASTP searches for similar proteins in the GenBank/EMBL and Swiss-Prot databases were performed with the expect threshold value increased from the default 10 to 30.

### Phylogenetic analysis

Bayesian phylogenetic analysis was performed using the L protein sequences of the viruses described herein and representative rhabdoviruses listed in Supplementary Table S1. Sequences were aligned using MUSCLE 3.6, and ambiguously aligned regions were trimmed away using Gblocks.^58,59^ Bayesian analysis was performed using BEAST,^60^ employing a WAG model of amino acid substitution with gamma + invariant site heterogeneity. A lognormal relaxed clock model was used with a tree prior set to coalescent:exponential growth. The model was run with a Markov chain Monte Carlo chain length of 40 000 000 with the output logged every 4000 steps producing 10 000 trees. The maximum clade credibility tree was chosen (1000 tree burn-in), and trees were edited using FigTree v1.4 (http://tree.bio.ed.ac.uk/software/figtree/).

## Results and Discussion

### Whole genome sequencing

HOJV was sequenced using traditional Sanger sequencing and the PCR-select cDNA subtraction method, producing 85% coverage of the genome. The remaining gaps between contigs were filled by sequencing of PCR products generated using virus-specific primers. The final consensus sequence had at least six times coverage. High-throughput sequencing of ORV on the Illumina GAIIx platform produced sequence for almost the entire genome with average coverage of 28 times (minimum coverage of five times). The genome termini of both viruses were subsequently obtained by the RACE method. The sequencing of the same isolate of ORV, excluding nine nucleotides (nt) at both the 3′ and 5′ termini, was concurrently performed by Walker et al.^7^ Comparison of the two sequences shows mismatches at nucleotide positions 5984 and 6104 in the G gene, resulting in R to K and I to S amino acid changes, respectively, and at position 12 177 within the L gene, resulting in an S to N amino acid change. As these changes are in areas that are generally not highly conserved, it is possible that they are a product of virus passage and adaptation to cell culture.

### Similarity

**Figure 1.**
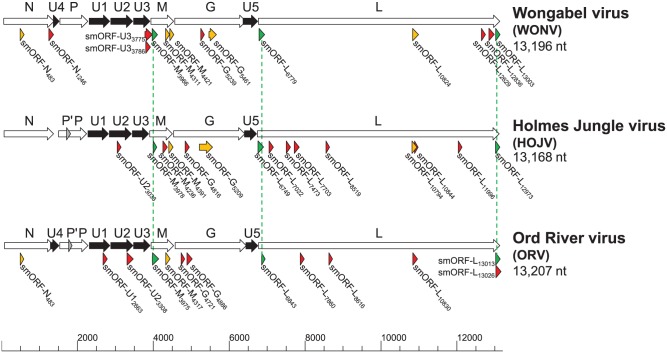
Genome maps of Holmes Jungle virus (HOJV), Ord River virus (ORV), and Wongabel virus (WONV). Arrows beneath the genomes depict small open reading frames (smORFs) ≥93 nt (30 aa) in length. smORFs that are conserved between all three viruses are illustrated by the green arrows and dotted lines, smORFs conserved between two of the viruses are depicted by the yellow arrows, and unpaired smORFs are depicted by the red arrows. The numerical values indicate the position of each smORF on the genome.

**Figure 2.**
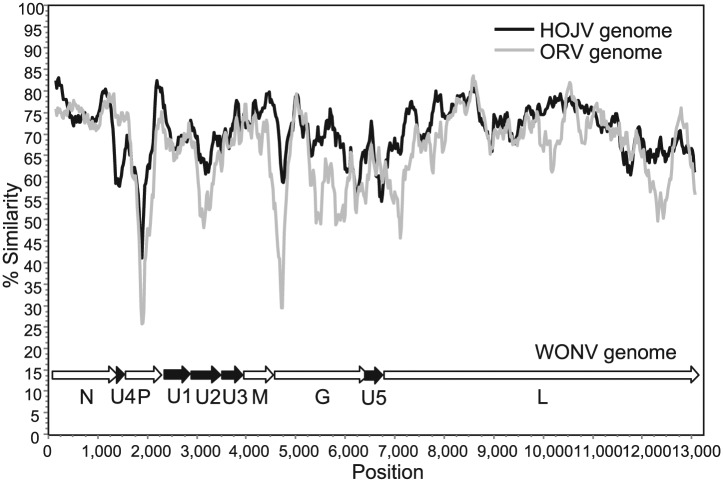
A similarity plot comparing the complete genomes of HOJV and ORV to the WONV genome (WONV genes represented below the plot by the arrows). Whole genomes were aligned by ClustalW, and pairwise identities were scanned using a window size of 300 nt, step of 20 nt, and the Kimura 2-parameter model. HOJV indicates Holmes Jungle virus; ORV, Ord River virus; WONV, Wongabel virus.

**Figure 3.**
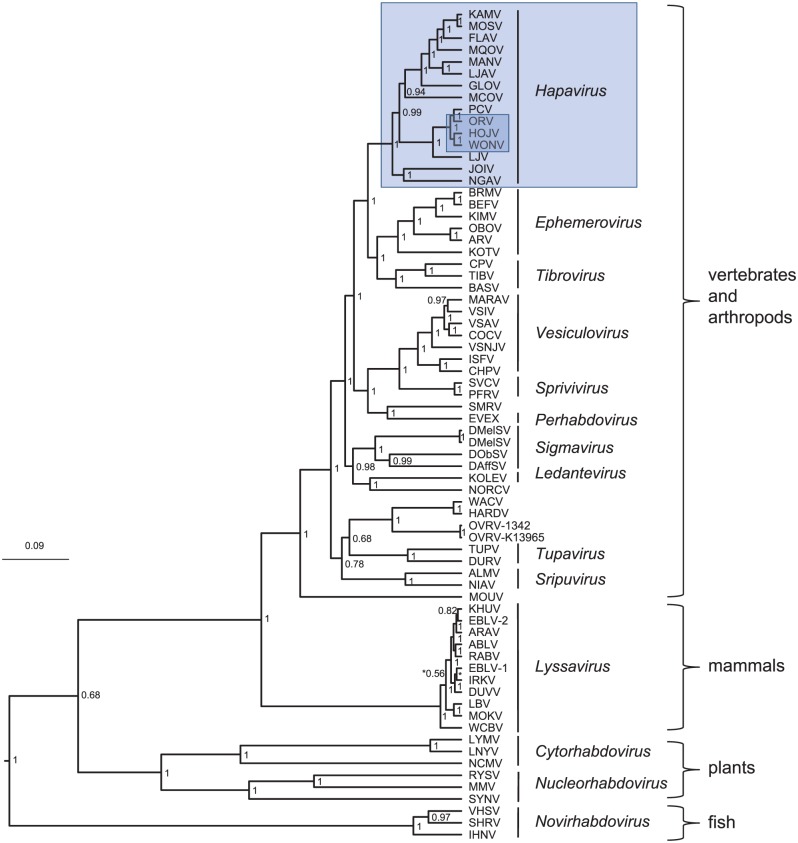
Bayesian phylogenetic analysis of HOJV with representative rhabdoviruses using a 1114-aa sequence of the L protein derived from Gblocks. Bayesian posterior probabilities are indicated. Genera are italicised, and unmarked viruses are currently unassigned by the International Committee on Taxonomy of Viruses. Bar represents amino acid substitutions per site. The genus *Hapavirus* and the three viruses analysed for smORFs in this study (HOJV, ORV, and WONV) are highlighted by the blue boxes. HOJV indicates Holmes Jungle virus; ORV, Ord River virus; WONV, Wongabel virus.

The completed genomes of HOJV and ORV are 13 168 and 13 207 nt, respectively (GenBank accession numbers KY421919 and KY421920, respectively). The two viruses share high overall sequence identity with WONV (Table 1) and share a similar genome structure, each containing three additional ORFs located between the P and M ORFs and a single ORF between the G and L ORFs (Figure 1). These viruses also demonstrate high conservation of transcription control sequences (Supplementary Table S2). Despite the high sequence identities, there are two notable differences between the three genomes: HOJV does not contain a U4 ORF overlapping the N gene that is present in WONV and ORV, and HOJV and ORV each contain a putative small P′ ORF overlapping the P gene, which is absent from WONV. A plot of genome similarities of HOJV and ORV plotted to WONV (Figure 2) illustrates that ORV is generally less similar to WONV than HOJV is, particularly at the highly diverged P, G and U2 ORFs, and at both ends of the L ORF. Bayesian phylogenetic analysis using a Gblocks-trimmed alignment of complete L proteins (Figure 3) suggests that the three viruses diverged relatively recently. The analysis demonstrates that HOJV and WONV form a clade, whereas ORV forms a clade with another recently sequenced Australian virus, Parry Creek virus (PCV).^7^

**Table 1.** Similarities of genome and protein sequences of HOJV, ORV, and WONV.

Nucleotide identity, %			
		ORV	WONV
Genome	HOJV	74	77
	ORV	—	75
Amino acid identity/**similarity**, %			
		ORV	WONV
N	HOJV	93/**97**	95/**97**
	ORV	—	93/**95**
U4	HOJV	NA	NA
	ORV	—	65/**88**^a^
P	HOJV	65/**79**	76/**86**
	ORV	—	64/**75**
U1	HOJV	81/**92**	87/**94**
	ORV	—	84/**92**
U2	HOJV	71/**85**	79/**93**
	ORV	—	76/**86**
U3	HOJV	79/**92**	86/**94**
	ORV	—	78/**92**
M	HOJV	81/**95**	87/**93**
	ORV	—	81/**92**
G	HOJV	68/**83**	83/**91**
	ORV	—	70/**85**
U5	HOJV	71/**87**	72/**88**^b^
	ORV	—	72/**89**^b^
L	HOJV	84/**93**	88/**95**
	ORV	—	84/**93**

Abbreviations: HOJV indicates Holmes Jungle virus; NA, not applicable; ORV, Ord River virus; WONV, Wongabel virus.

The above global sequence alignments were performed using EMBOSS Needle algorithm.

a Alignment of the 49-aa equivalent region of ORV U4 (complete length: 75 aa) with the complete 49-aa WONV U4 protein.

b Alignment of the 106-aa equivalent region of the WONV U5 protein (complete length: 127 aa) with the complete HOJV and ORV U5 proteins.

### Antigenic relationships

Previously published serological cross-reactivity studies between various Australian rhabdoviruses showed significant one-way neutralisation of HOJV by WONV antiserum (titre: 256; homologous titre: 1280).^56^ Reciprocal neutralisation was not observed; however, this may have been due to the low homologous neutralisation titre (80) of the HOJV antiserum. Antibodies generated to HOJV and WONV also did not appear to significantly cross-neutralise ORV. In the absence of suitable antibodies to ORV, reciprocal comparisons could not be made. Although the antigenic picture is not yet complete, the level of observed cross-reactivity suggests that these three viruses are different at the antigenic level and constitute separate species. Antigenic comparisons with other members of this genus, particularly those that have distant geographical origins and have never been directly compared, would be important to inform the demarcation of species within this genus. An understanding of the antigenic cross-reactivities between the different viruses might also prove valuable to clinical diagnostics in the event of any suspected human or animal disease cases.

### Phylogenetic analysis and taxonomy

Phylogenetic analysis demonstrates that HOJV belongs to a clade that currently contains the Australian viruses WONV, ORV, and PCV (Figure 3). Another Australian virus, Little Lilly Creek virus (LLCV, OR559), is also likely to be placed into this clade, as indicated by a short, highly conserved 195-nt fragment that has been produced for the L gene using a consensus PCR approach (Julian Druce, Victorian Infectious Diseases Reference Laboratory, Melbourne, personal communication, 2014). LLCV was isolated during the same study that isolated ORV and PCV.^26,61^ This fragment displays 96% identity with ORV and 74% to 75% identity with HOJV, WONV and PCV. Unpublished historical information suggests that LLCV is antigenically distinct from ORV, although definitive serological cross-reactivity studies and further sequence data are required to confirm that it is a separate species.

The new genus *Hapavirus* currently contains 15 species.^2^ Previous maximum likelihood phylogenetic analysis of the genus suggests that the HOJV/ORV/WONV/PCV clade forms one of two distinct subgroups within this genus.^7^ Our Bayesian phylogenetic analysis, however, suggests that there may be three subgroups formed, with NGAV and JOIV forming a distant third branch (Figure 3). This demonstrates that the relationships between species within the genus still need to be confirmed. It is important to note that the characterisation of new members in the future will probably influence the currently perceived relationships and dynamics within the genus. There are at least two other candidate hapaviruses that await further characterisation: Bangoran virus (BGNV) isolated from *Culex* mosquitoes and from the brain of a bird from Africa, and Porton virus (PORV) isolated from *Mansonia* mosquitoes from Malaysia. Although BGNV and PORV have previously tested negative for antigenic cross-reactivity with other hapaviruses,^17^ partial sequence data suggest that they are members of this genus.^18^ Characterisation of additional members, such as BGNV and PORV, will be important in shaping the demarcation criteria for species within the genus. The presence of accessory genes to date has played a major role in species demarcation (eg, presence of G_NS_ in ephemeroviruses). It is currently uncertain whether the presence of accessory ORFs will play a role in species demarcation within the genus *Hapavirus*, which has some of the greatest accessory ORF diversity observed within the family *Rhabdoviridae*. As evidence increases to suggest that the likely frequent gain or loss of accessory genes in rhabdoviruses has played a significant role in rhabdovirus evolution,^7^ the extent to which these additional genes should play a role in taxonomy is still challenging. This is compounded by an insufficient understanding of the mechanisms involved in the creation of additional genes in rhabdoviruses (ie, gene duplication, or homologous or lateral recombination).^7,62–64^

### Structural proteins N, P, M, G, and L

Detailed analyses of the WONV N, P, M, G and L proteins were previously described.^6^ The cognate HOJV and ORV proteins share high overall sequence similarity with those of WONV, with highest similarity observed among the N proteins (95%-97%) and the lowest among the P proteins (75%-86%) (Table 1). Comparisons of the HOJV and ORV structural proteins with those of WONV showed an overall conservation of all the key motifs previously identified in WONV with no remarkable differences observed (results not shown). One prominent observation was made in relation to the presence of an additional five conserved in-frame methionine residues in the M proteins of all three viruses, which suggests that truncated M protein products might also be generated via polycistronic transcription and translation (Supplementary Figure S1). The presence of the first two methionine residues, at positions 57 and 106, suggests the production of shorter translation products, which are comparable in size with, and may have similar function as the M2 and M3 products of vesicular stomatitis virus (VSV) known to have an important role in viral cytopathology.^65^ The other three conserved in-frame methionine residues are near the carboxy-terminus of the M protein at residues 170, 180, and 192 in all three viruses, which would result in very short translated products of <30 aa in length. In consideration of the multifunctional role of the rhabdovirus M protein in virion assembly, budding and pathogenesis, together with the known significance of the VSV M2 and M3 products, it is reasonable to speculate that these shorter in-frame M translation products conserved in all three viruses may have functional roles and warrant further study. This observation also underscores the hypothesis that rhabdovirus genomes are capable of coding for additional small proteins that are currently unrecognised.

### Accessory proteins U1, U2, and U3

The roles of the putative accessory U1, U2 and U3 proteins of hapaviruses are still largely unknown. Studies suggest that the WONV U3 protein may be involved in redirecting or blocking the insect response to infection by binding to the ‘inhibition of apoptosis protein apollon’ and a component of the chromatin remodelling complex.^64^ It has been observed previously that the U1, U2 and U3 proteins of WONV, FLAV and HPV share more similarity with each other than they do with any other known proteins.^3,64^ It is suggested that the high level of similarity and significant ‘signals for paralogy’ between these proteins may indicate that their genes arose through the process of gene duplication in an ancestor, which is also the proposed mechanism for the existence of the additional glycoprotein (G_NS_) and adjacent accessory ORFs in ephemeroviruses.^43,63,64^ The theory of duplication as a widely used mechanism for the emergence of new ORFs is further supported by recent observations in other rhabdoviruses.^7^

The WONV U1 protein is approximately 60% similar to its U2 protein and 57% to its U3 protein, whereas the U2 and U3 proteins share considerably lower sequence similarity (31%).^64^ A similar pattern is observed in HOJV and ORV; however, in each virus, the U1 protein has somewhat higher similarity with the U3 proteins (58% in both viruses) than the U2 proteins (55.5% and 55%, respectively). The level of similarity between the U2 and U3 proteins of HOJV and ORV is 33% and 27%, respectively. The significance of these similarities is unclear but it does support the theory that the order of duplication of the U1, U2, and U3 genes did not occur sequentially.^3^ To further support this, the central regions of the U1 and U3 proteins of all three viruses contain the previously observed common sequence (KSxYDFVWPxxxxLxxG), which interestingly is not present in the U2 proteins.^64^ It is possible that each protein has a similar function but is used in different circumstances (eg, in different hosts). An alternate upstream start signal in the U1 proteins of all three viruses suggests that a longer protein by 11 residues could also be produced, although translation of this variant would consequently need to occur from a bicistronic P-U1 mRNA.

### Accessory protein U4

**Figure 4.**
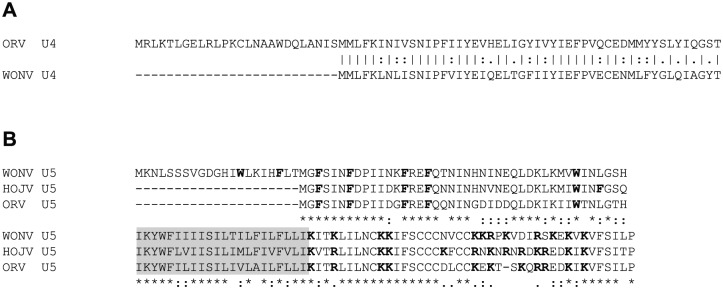
(A) Global (EMBOSS Needle) pairwise alignment of the putative U4 proteins of ORV and WONV encoded by an ORF overlapping the N gene. The homologous 49-aa region of the 75-aa ORV U4 demonstrates 88% amino acid similarity to the 49-aa WONV U4. (B) MUSCLE alignment of the U5 proteins (hypothetical viroporins) of WONV, HOJV, and ORV. Fully conserved (*), strongly conserved (:), and weakly conserved (.) amino acids are marked. Predicted transmembrane regions are shaded. Aromatic residues (F and W) in the N-terminal domain and basic residues (K and R) within the C-terminal domain are given in bold. Six conserved cysteine residues in the C-terminal domains are marked by the boxes. HOJV indicates Holmes Jungle virus; ORV, Ord River virus; WONV, Wongabel virus.

The WONV U4 ORF, which overlaps the 3′ untranslated region (UTR) of the N gene, was previously described to putatively code for a small 49-aa polypeptide.^6^ Ord River virus contains a considerably larger ORF of 228 nt (75 aa) in the corresponding location; however, the equivalent 49-aa region of the ORV U4 protein (from the second in-frame methionine residue at position 27) shares 88% amino acid similarity (73% nucleotide identity) with the WONV U4 protein (Figure 4A). The two polypeptides share similar predicted topology, both containing a tentative transmembrane-like region predicted by the PHDhtm algorithm but not corroborated by the other algorithms used. Interestingly, the equivalent regions in HOJV and FLAV do not contain a U4 ORF, suggesting a non-essential role or one that is possibly fulfilled by a protein coded elsewhere on the genome.

### Accessory protein U5

The U5 proteins of WONV, HOJV and ORV resemble viroporins based on predicted secondary structure similarities to, and similar residue content of, the α1 viroporin of ephemeroviruses and predicted viroporins in other rhabdoviruses encoded between the G and L ORFs.^6,7,41,45,66,67^ Studies of the α1 viroporin of bovine ephemeral fever virus (BEFV) demonstrate that it localises to the Golgi complex, and the C-terminal cytoplasmic region of the protein contains a strong nuclear localisation signal that translocates to the nucleus.^67^ Its interaction with importins suggests a role in modulating nuclear trafficking pathways. The WONV U5 ORF is similarly located within the UTR that follows the G ORF and contains three alternate in-frame start codons resulting in putative products of 127, 106 or 73 aa.^6^ HOJV and ORV contain comparable ORFs that putatively encode proteins of 106 and 105 aa, respectively, suggesting that the 106-aa product of WONV is the likely one to be produced. The HOJV, ORV and WONV U5 proteins share 62% amino acid sequence identity and 88% similarity, and demonstrate conservation of the viroporin-like characteristics previously observed in WONV (Figure 4B). In contrast to the BEFV α1 protein, the U5 proteins of WONV, HOJV and ORV are not predicted to contain nuclear localisation signals; therefore, an analogous role in nuclear trafficking is unlikely.

### Polycistronic transcription and putative smORFs in HOJV, ORV, and WONV

In rhabdoviruses, most of the structural protein ORFs (ie, N, P, M, G, and L) contain autonomous conserved transcription initiation and termination signals. These signals dictate the monocistronic mode of transcription that is typical of −ssRNA viruses. Many of the large accessory protein genes of hapaviruses (eg, U1, U2 and U3) also appear to contain autonomous transcription signals; however, in some cases, these signals appear to be less conserved and thus may be leaky. This feature, along with a flexible RNA polymerase, is believed to bestow rhabdoviruses with an ability to produce various polycistronic mRNA transcripts that contain multiple ORFs. It is believed that polycistronic transcription is a useful means to maximise coding capacity within relatively compact genomes, as well as to optimise and control production of non-essential accessory proteins.^64^ The most widely recognised examples of the use of the polycistronic mechanism is the C ORF (also known as P′) present in numerous rhabdoviruses overlapping the P protein and the accessory proteins of ephemeroviruses located between the G and L genes. Multiple variants of polycistronic transcripts have also been observed in a highly passaged strain of rabies virus (which is traditionally a monocistronic virus) demonstrating the adaptability and flexibility of the rhabdoviral polymerase.^68^ In HOJV, ORV and WONV it appears that the transcription of the U4 and U5 ORFs can only be possible via polycistronic mRNAs that contain the N and G ORFs, respectively, due to an apparent absence of independent transcription machinery. The mechanism for translation from polycistronic mRNAs is still not well-understood but may include internal ribosomal initiation, leaky ribosomal scanning and ribosomal frameshifting (−1) to produce a polyprotein, or coupled translation.^3,69,70^ Further evidence for some of these mechanisms in rhabdoviruses has recently been proposed.^7^

**Figure 5.**
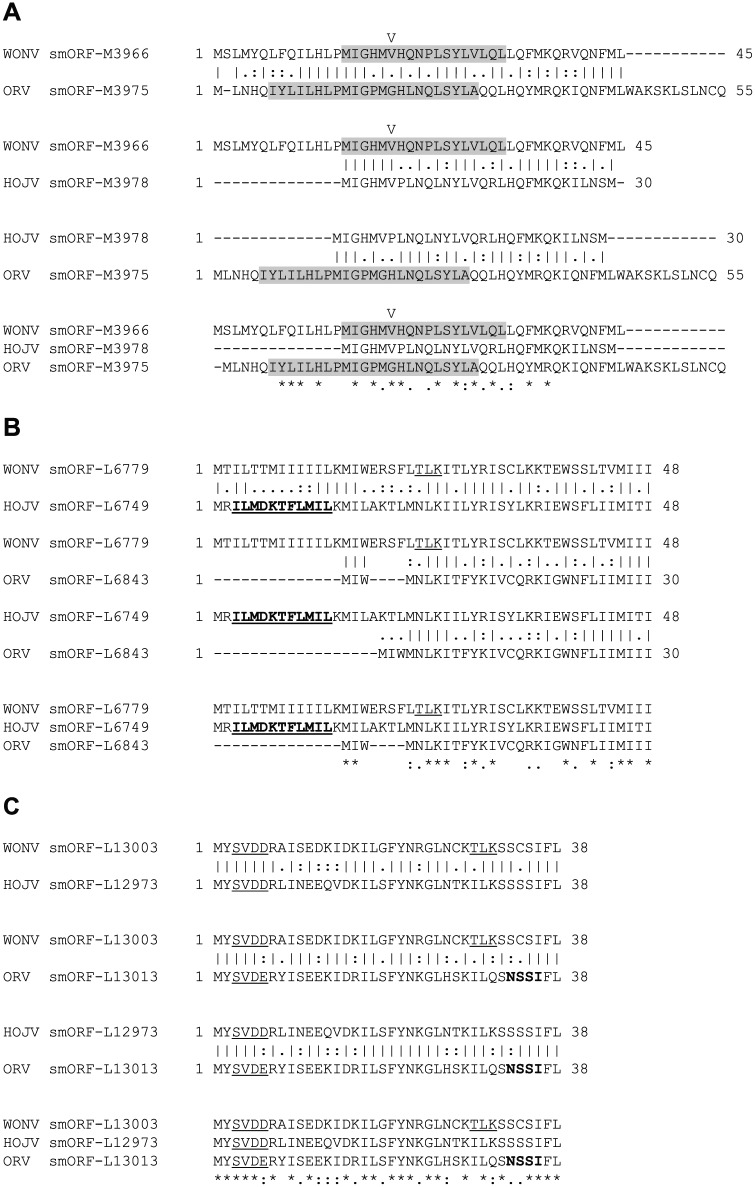
Global pairwise (EMBOSS Needle) and multiple (MUSCLE) sequence alignments demonstrating similarities between the three sets of smORF protein products that are conserved in all three viruses (WONV, HOJV and ORV). Numerical values indicate location on the genome. (A) Conserved smORF located within the M gene of all three viruses. Predicted transmembrane regions are shaded and a potential signal peptide cleavage site (V) in the WONV smORF is indicated. (B) Conserved smORF located near the start of the L ORF in all three viruses. The predicted ‘serpins signature’ in the HOJV smORF-L_6749_ protein is given in bold and underlined. Predicted phosphorylation sites are underlined. (C) Conserved smORF located near the end of the L ORF in all three viruses. Predicted glycosylation sites are given in bold and phosphorylation sites are underlined. HOJV indicates Holmes Jungle virus; ORV, Ord River virus; smORF, small open reading frame; WONV, Wongabel virus.

**Figure 6.**
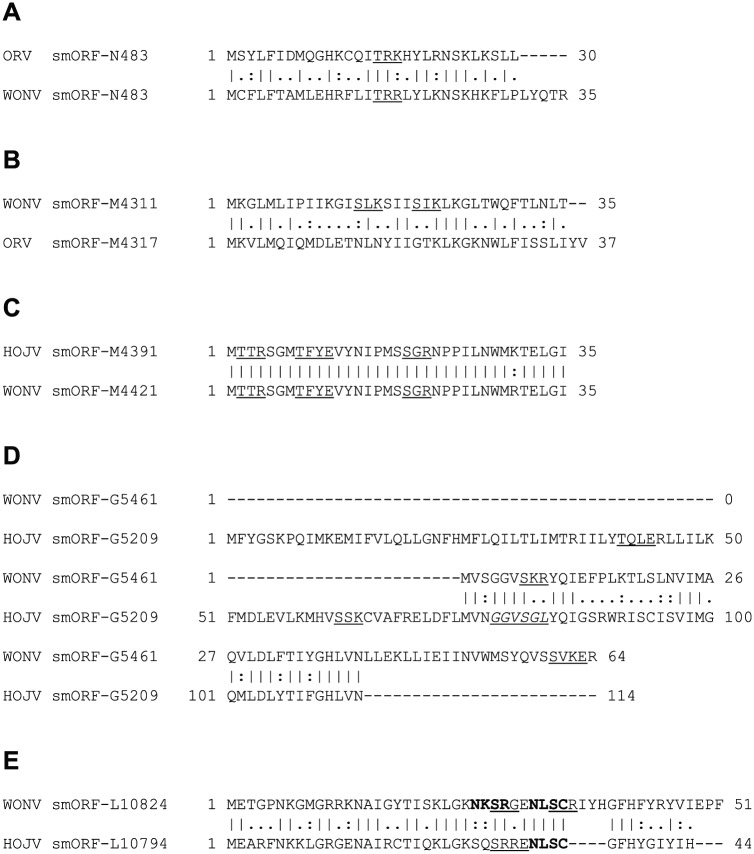
Global pairwise (EMBOSS Needle) alignments demonstrating similarities between smORF protein products that are conserved between only two viruses. Numerical values indicate location on the genome. Predicted glycosylation sites are given in bold, phosphorylation sites are underlined, and myristoylation sites are italicised and underlined. (A) Conserved smORF protein product located within the N ORF of ORV and WONV. (B) Conserved smORF protein product located within the M ORF of ORV and WONV. (C) Conserved smORF protein product located within the M ORF of HOJV and WONV, noting that the sequences differ by just one residue. (D) Conserved smORF protein product located within the G ORF of WONV and HOJV. (E) Conserved smORF protein product located within the L ORFs of WONV and HOJV containing a cluster of predicted glycosylation and phosphorylation sites. HOJV indicates Holmes Jungle virus; ORV, Ord River virus; smORF, small open reading frame; WONV, Wongabel virus.

The genomes of HOJV, ORV and WONV contain an abundance of very small predicted ORFs located in various areas of the genome, interlaced within the larger ORFs. On the basis of the discernible presence of polycistronic transcription and translation mechanisms, it is plausible that these smORFs could encode functional proteins. Predictive analysis of the three viral genomes for smORFs coding for proteins with an arbitrarily selected 30-aa size cut-off limit (with the aim of producing ORFs of a sufficient size that could be meaningfully analysed) resulted in the identification of 14 smORFs in WONV, 15 in HOJV and 13 in ORV (Figure 1). The greatest abundance of smORFs in all three viruses appears to be around the highly diverged M and G genes and in regions proximal to the start and end of the L ORF. Three smORFs appear to be conserved between all three viruses (Table 2, Figure 5): one within M, one near the start of L, and one near the end of L ORFs. Another five smORFs appear to be conserved between two of the viruses (Figure 6): within the N, M, G, and L ORFs. All of the matched smORF protein products display high sequence similarity; several also have similar predicted structural features and post-translational modification sites (Figures 5 and 6). Some of the observed smORF proteins also have features that may be indicative of function. For example, the 48-aa HOJV smORF-L_6749_ contains a characteristic 10-residue ‘serpins signature’, which is commonly found in serine protease inhibitors that play a role in modulation of blood coagulation and inflammation. This signature is also found in carriage and storage proteins, but, moreover, some viruses, such as poxviruses, are known to produce serpins to help evade host immune defences.^71^ Interestingly, this signature is not present in the equivalent ORV and WONV smORFs, which might be due to a non-essential role or adaptation to a different host. Another example is the 54-aa ORV smORF-U2_3308_ which contains a characteristic leucine zipper motif (LxxxxxxLxxxxxxLxxxxxxL) (located at residues 4-25), which may be indicative of a role in transcription. The N-terminal region of the protein (residues 1-6) is also predicted with high confidence to be protein binding, which could also suggest another function.

**Table 2.** Putative smORFs of HOJV, ORV, and WONV.

smORF name	Position, nt	Location	Length, nt	Length, aa	MW, kDa	pI
smORFs present in all 3 viruses						
WONV smORF-M_3966_	3966-4103	M	138	45	5.5	10.0
HOJV smORF-M_3978_	3978-4070	M	93	30	3.6	10.7
ORV smORF-M_3975_	3975-4142	M	168	55	6.6	10.0
WONV smORF-L_6779_	6779-6925	L	147	48	5.7	10.3
HOJV smORF-L_6749_	6749-6895	L	147	48	5.9	10.8
ORV smORF-L_6843_	6843-6935	L	93	30	3.7	10.5
WONV smORF-L_13003_	13 003-13 779	L	117	38	4.3	8.3
HOJV smORF-L_12973_	12 973-13 089	L	117	38	4.4	7.0
ORV smORF-L_13013_	13 013-13 129	L	120	39	4.6	10.5
smORFs present in 2 viruses						
WONV smORF-N_483_	483-590	N	108	35	4.4	10.9
ORV smORF-N_483_	483-575	N	93	30	3.7	10.4
WONV smORF-M_4311_	4311-4418	M	108	35	3.9	11.1
ORV smORF-M_4317_	4317-4430	M	114	37	4.4	9.8
WONV smORF-M_4421_	4421-4528	M	108	35	4.1	9.6
HOJV smORF-M_4391_	4391-4498	M	108	35	4.0	9.5
WONV smORF-G_5461_	5461-5655	G	195	64	7.4	9.3
HOJV smORF-G_5209_	5209-5553	G	345	114	13.3	9.3
WONV smORF-L_10824_	10 824-10 979	L	156	51	5.9	10.4
HOJV smORF-L_10794_	10 794-10 928	L	135	44	5.1	10.4

Abbreviations: HOJV indicates Holmes Jungle virus; MW, molecular weight; ORV, Ord River virus; smORF, small open reading frame; WONV, Wongabel virus.

Most of the identified smORFs in this study are located within the structural protein ORFs; they do not appear to contain autonomous consensus transcription control sequences and thus would seem to rely on the polycistronic mechanism for translation; however, there are a few exceptions. The transcription of WONV smORF-N_1246_ could potentially be initiated via a signal that resembles a conserved transcription start signal (AGCAG) located 108 nt upstream, with termination occurring at the same transcription stop signal that is used by the N gene. Similarly, transcription of the WONV smORF-U3_3775_ may be initiated via a possible start signal (AGTAG) located 80 nt upstream, which could furthermore result in transcription of a polycistronic mRNA comprising smORF-U3_3775_, the M ORF and the three other smORFs that overlap the M ORF. It is possible that transcription of some of the remaining smORFs may be directed by transcription start signals that are less conserved and thus difficult to identify.

All three viruses contain some smORFs that do not have observable similarities with others despite being located in similar regions of the genome. Many of these unpaired smORFs, particularly those coding for polypeptides <40 aa in size, are unremarkable in their features, although a substantial number do have significant (>40%) similarity to regions in various known or hypothetical proteins, which may be indicative of functional domains.

A precedent for the presence of smORFs is set by observations in some plant, fungus and insect viruses from other families.^52–54^ We thus speculate that the identified smORFs in this study may have similar roles in the arthropod vector; however, to support this view, methodical analyses would be required of other rhabdoviruses, including those that do not seem to rely on arthropod transmission and contain smaller and simpler genomes, such as the lyssaviruses and vesiculoviruses. However, it is also possible that smORFs may have a role in the vertebrate host, for example, in suppression of the immune response or regulation of other host factors in favour of infection. If this is the case, a greater understanding of the roles of smORFs in −ssRNA viruses could offer potential new targets for exploitation in the development of prophylactic or therapeutic anti-viral strategies or other health applications.

### Necessity of characterising novel arboviruses

**Figure 7.**
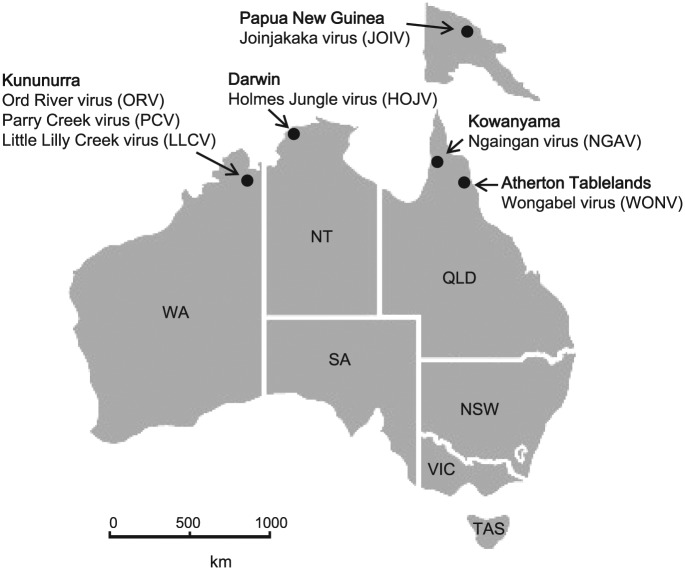
Map of Australia, including Papua New Guinea, highlighting the locations of isolation of viruses of the genus *Hapavirus* from the region. HOJV, ORV, PCV, and LLCV were isolated from *Culex annulirostris* mosquitoes; JOIV was isolated from a mixed pool of culicine mosquitoes; WONV and NGAV were isolated from biting midges *Culicoides austropalpalis* and *Culicoides brevitarsis*/*actoni*, respectively. HOJV indicates Holmes Jungle virus; JOIV, Joinjakaka virus; LLCV, Little Lilly Creek virus; NGAV, Ngaingan virus; ORV, Ord River virus; PCV, Parry Creek virus; WONV, Wongabel virus.

HOJV, ORV, PCV and LLCV were all isolated from the *C. annulirostris* mosquito from Northern Australia (Figure 7). Although WONV has been isolated from biting midges (on one occasion only), the above trend suggests that mosquitoes may also be involved in the transmission of WONV. Knowledge of the type of insect vector is critical from the perspectives of disease transmission, pathology and epidemiology. The two types of insects feed quite differently; midges are pool feeders, whereas mosquitoes feed directly from the venule, thus transmitted viruses presumably bypass the lymphatic system (T. D. St George, personal communication, 2014). Furthermore, the questions of whether these viruses infect the same host or whether each is adapted to a different host are intriguing and deserve further investigation. The vertebrate hosts of these viruses are largely unknown other than the equivocal indication that WONV infects birds. LJV isolated from *Culex dunni* mosquitoes in Panama, with a suspected rodent host, is the only other virus known to belong to this clade. The arid and tropical regions of Northern Australia are abundant in different species of native and introduced rodents, as well as a variety of endemic marsupials and bats (insectivorous and fruit) that could provide unique niches for the evolution viruses, such as WONV, HOJV, ORV, PCV and LLCV.

The importance of a comprehensive understanding of the different groups of viruses within this family is emphasised by the recent emergence of BASV in Africa.^44^ BASV was identified to belong to the genus *Tibrovirus* known to only consist of asymptomatic viruses of cattle (TIBV, CPV, Bivens Arm and Sweetwater Branch viruses) present throughout the top half of Australia, Southeast Asia and the Americas.^45,72,73^ Although BASV initially caused concern due to its suspected role in human haemorrhagic disease, the available information on these tibroviruses abetted a scrutinous analysis of this virus. Subsequent identification of two additional tibroviruses, Ekpoma virus-1 and Ekpoma virus-2, in apparently healthy individuals, with a high seroprevalence (45%) in tested individuals in Nigeria, further suggests that the link to disease may be tenuous.^74^

It is imperative, however, to understand the potential consequences on disease severity of pre-existing antibodies from exposure to related rhabdoviruses. Naturally acquired BEFV infections sometimes result in prolonged paralysis compared with the much shorter duration of symptoms in experimentally infected cattle. It is speculated that the pre-existence of heterotypic antibodies from previous infections with related ephemeroviruses, such as Kimberley or Berrimah viruses that circulate asymptomatically in cattle in the region, could contribute to the development of these symptoms (T. D. St George, personal communication, 2016). This concept is comparable, for example, with the dengue antibody-dependent enhancement of symptoms following infection with different serotypes of the virus.

The potential influence of this group of viruses on replication of other important local viruses (eg, BEFV or viruses from other families such as Ross River virus) in the insect vector could also be useful. Some insect viruses are capable of suppressing or enhancing the replication of other viruses, for example, the recently described Palm Creek flavivirus (also isolated from Northern Australia) which was found to suppress West Nile virus and Murray Valley encephalitis virus replication in mosquito cells.^75^ In consideration of the observation that the Australian hapaviruses are widely distributed across Northern Australia (Figure 7), further investigations would be useful into the potential roles and risks associated with these viruses in the regulation of transmission of other medically or veterinary important viruses.

### Concluding remarks

The links to human infection by some members of the genus *Hapavirus* and the wide range of other hosts that these viruses infect (birds, primates, marsupials, livestock, rodents, reptiles, and arthropods) are significant, and thus, this group merits further investigation. From the Australian perspective, the *C. annulirostris* mosquito is involved in the transmission of several arboviruses of concern to human and animal health. Thus, it is important to understand the types of viruses, such as HOJV, ORV, PCV and LLCV that are carried by this vector. It would be useful to perform wider-ranging serosurveys to more accurately define the range of vertebrate and invertebrate hosts and to assess the potential health risks. The reported study highlights that it is important to further analyse and understand the complexities of the rhabdovirus genome, including the roles of the smORFs herein identified in HOJV, ORV and WONV, as well as their occurrence in other rhabdoviruses and other −ssRNA virus families such as the *Filoviridae* and *Paramyxoviridae*.

## Supplementary Material

Supplementary material
