# Supplementary material for: Identification of very small open reading frames in the genomes of Holmes Jungle virus, Ord River virus, and Wongabel virus of the genus Hapavirus, family Rhabdoviridae
Source: Evol Bioinform Online. 2017 Jul 11;13:1176934317713484. doi: 10.1177/1176934317713484 (PMC5510769; doi:10.1177/1176934317713484)
Supplement: Supplementary material [file f_SupplementaryData_713484_8948.pdf]

SUPPLEMENTARY ONLINE DATA

Supplementary Table S1: Viruses and GenBank accession numbers used in phylogenetic analysis

VIRUS	ABBREVIATION	GENBANK ACCESSION
Almpiwar virus	ALMV	NC_025391.1
Australian bat lyssavirus	ABLV	NC_003243.1
Aravan virus	ARAV	EF614259.1
Adelaide River virus	ARV	JN935380.1
Bas-Congo virus	BASV	JX297815.1
Berrimah virus	BRMV	HM461974.1
Bovine ephemeral fever virus	BEFV	NC_002526.1
Chandipura virus	CHPV	AJ810083.1
Coastal Plains virus	CPV	GQ294473.1
Cocal virus	COCV	EU373657.1
Drosophilia affinis sigma virus	DAffSV	GQ410980.1
Drosophilia melanogaster sigma virus AP30	DmelSV AP30	NC_013135.1
Drosophilia melanogaster sigma virus HAP23	DMelSV HAP23	GQ375258.1
Drosophilia obscura sigma virus	DObsSV	GQ410979.1
Durham virus	DURV	FJ952155.1
Duvenhage virus	DUVV	NC_020810.1
European bat lyssavirus 1	EBLV1	NC_009527.1
European bat lyssavirus 2	EBLV2	NC_009528.1
Eel virus European X	EVEX	FN557213.1
Flanders virus	FLAV	AF523199.1
Gray Lodge virus	GLOV	AJR28572
Harrison Dam virus	HARDV	KJ432573.1
Infectious haematopoietic necrosis virus	IHNV	X89213
Irkut virus	IRKV	EF614260.1
Isfahan virus	ISFV	AJ810084.2
Joinjakaka virus	JOIV	AJR28531
Kamese virus	KAMV	AJR28326
Khujand lyssavirus	KHUV	EF614261.1
Kimberley virus	KIMV	JQ941664.1
Kolente virus	KOLEV	KC984953
Kotonkan virus	KOTV	HM474855.1
La Joya virus	LJV	AJR28296
Lagos Bat virus	LBV	EU259198.1
Landjia virus	LJAV	AJR28479
Lettuce necrotic yellows rhabdovirus	LNYV	NC_007642.1
Lettuce yellow mottle virus	LYMV	NC_011532.1
Manitoba virus	MANV	AJR28463
Maraba virus	MARAV	HQ660076.1
Marco virus	MCOV	AJR28445
Maize mosaic virus	MMV	NC_005975
Mokola virus	MOKV	NC_006429.1
Mosqueiro virus	MQOV	AJR28515
Mossuril virus	MOSV	AJR28353
Moussa virus	MOUV	FJ985748.1
North cereal mosaic virus	NCMV	NC_002251
Ngaingan virus	NGAV	NC_013955.1
Niakha virus	NIAV	KC585008.1
North Creek virus	NORCV	KF36097
Oak Vale virus 1342	OVRV 1342	JF705876.1

Oak Vale virus K13965	OVRV K13965	JF705877.1
Obodhiang virus	OBOV	HM856902.1
Parry Creek virus	PCRV	AJR28317
Pike Fry rhabdovirus	PFRV	FJ872827.1
Rabies virus	RABV	NC_001542.1
Rice yellow stunt virus	RYSV	NC_003746
Scophthalmus maximus rhabdovirus	SMRV	HQ003891.1
Snakehead rhabdovirus	SHRV	NC_000903.1
Sonchus yellow net virus	SYNV	NC_001615
Spring viremia of carp virus	SVCV	NC_002803.1
Tibrogargan virus	TIBV	GQ294472.1
Tupaia virus	TUPV	NC_007020.1
Viral haemorrhagic septicaemia virus	VHSV	NC_000855
Vesicular stomatitis Alagoas virus	VSAV	EU373658.1
Vesicular stomatitis virus Indiana	VSIV	AF473864.1
Vesicular stomatitis virus New Jersey	VSNJV	M20166.1
Walkabout Creek virus	WACV	NC_028232.1
West Caucasian bat virus	WCBV	EF614258.1
Wongabel virus	WONV	NC_011639.1

Supplementary Table S2: HOJV and ORRV transcription and replication control sequences

Junction		Gene End	IGR	Gene Start
leader – N(U4)	HOJV	n.a.	n.a.	AGTAGTTCTTATGACTACATCTCAAATCAACAATG
	ORRV	n.a.	n.a.	AGTAGTTCTTATGACTACTTAACCAAGCAAAATG
	WONV	n.a.	n.a.	AGTAGTTTTCATGACTACATCTCCAAAAACAATG
N(U4) – P	HOJV*	TAA…104 nt…CATGAAAAAAA	TC	AGTAGTTATCATG
	ORRV	TAA…131 nt…CATGAAAAAAA	TC	AGTAGTTATTATG
	WONV	TAA…131 nt…CATGAAAAAAA	TC	AGTAGTTATCATG
P – U1	HOJV	TAAACCATGAAAAAAA	TC	AGTAGACATCTAGTCTACAAGATG
	ORRV	TAAAACATGAAAAAAA	TC	AGTAGATATCATATCTACACCATG
	WONV	TGATCACATGAAAAAAA	TC	AGTAGACATCTAGTCTACAAGATG
U1 – U2	HOJV	TGATCATGAAAAAAA	TC	GGCAGTCATCATG
	ORRV	TAGATCATGAAAAAAA	TC	GGCAGTCATCATG
	WONV	TAATACATGAAAAAAA	TC	GGCAGTCATCATG
U2 – U3	HOJV	TAAATCATGAAAAAAA	TC	AGTAGCAATCATG
	ORRV	CATGAAAAAAA	TT	AGTAGACATCATG
	WONV	TAAATCTTGAAAAAAA	TC	AGTAGTCATCATG
U3 – M	HOJV	TGATCATGAAAAAAA	CT	AGCAGAAATCATG
	ORRV	TGACACATGAAAAAAA	TT	AGCAGCAATCATG
	WONV	TAAATACATGAAAAAAA	TC	AGCAGTCATCATG
M – G(U5)	HOJV	TAACATGAAAAAAA	CC	AGTAGACATATTGAATACAAAATG
	ORRV	TAACATGAAAAAAA	TT	AGTAGACATATTGTCTAATTTTTTGAAAATG
	WONV	TAACATGAAAAAAA	CT	AGTAGACATATTGATTACAAGATG
G(U5) – L	HOJV	CATGAAAAAAA	CC	AGTAGGCATTATG
	ORRV	CATGAAAAAAA	CT	AGTAGACATAATG
	WONV	CTTGAAAAAAA	CT	AGCAGTCATAATG
L – trailer	HOJV	TAA…29 nt…CATGAAAAAAA	n.a.	n.a
	ORRV	TAA…29 nt…CATGAAAAAAA	n.a.	n.a.
	WONV	TAA…28 nt…CATGAAAAAAA	n.a.	n.a.

Stop and start codons are bolded and underlined, putative transcription start signals are bolded, and the transcription stop/polyadenylation signals are underlined. IGR, intergenic region. Remnant/non-functional or alternate translation start signals in the HOJV and ORRV N proteins, and a premature stop signal in the ORRV N protein are in grey type. (*) HOJV does not contain a U4 ORF. In ORRV and WONV, the first marked stop codon relates to the N ORF and the second marked stop codon (which overlaps the transcription stop signal in both viruses) relates to the U4 ORF. Brackets indicate overlapping ORFs.

```
ORRV      MLKIWRKKGKANYAQSSNLSDTTSPYDWAYGSSEPIELFSPTAPPVYETKNSKFHVMGQI
WONV      MLKIWRKKGRKHESDVSTVSDTSSPYDWAYGTSEPIELFSPTAPPVYETKSSKFHVMSQL
HOJV      MLKIWRKKGKGAGSTSSNLSETTSPYDWAYGASEPIKLFSPTAPPVYETKNSKFHVMCQL
          *********.   :  *.:*:*:********:****:*************.****** *:

ORRV      KIVTKLSIDSPEMLCQVLEQIVLKYNGSFKFKDHHLLNLILVGTHMKRELDHDTYVYKGL
WONV      KIATKLSIGSAEILCQILEQIVQKYNGSFKFKSHHLLNLILVGTHMKKELDHDSFIYKGL
HOJV      RIVTKLSIENAEILCQILEQIVQKYNGSFKYKNHHLMNLILVGTHMKKELDHDSFIYKGL
          .*.***** ..*:***:***** *******:*.***:**********.*****:::****

ORRV      WDDVIVYEGIDADTNGFGNKFELYHRYKIKGQELAVHFESDLRLTTRSGMTYYEVYNIPM
WONV      WDDVILYEGLDVDTNNQGNKFEIYHKYKIKGFDMAIHFESDLRMTTRSGMTFYEVYNIPM
HOJV      WDDVILYEGLDADTNVPGNKFEIYHKYKIKGYDMAIHFESDLRMTTRSGMTFYEVYNIPM
          *****:***:*.***  *****:**.***** ::*:*******:*******:********

ORRV      SSGRNPPILNWMRTELGI
WONV      SSGRNPPILNWMRTELGI
HOJV      SSGRNPPILNWMKTELGI
          ************.*****
```

Supplementary Figure S1: Multiple (MUSCLE) sequence alignment of the M proteins of HOJV, ORRV and WONV. Fully conserved (*), strongly conserved (:) and weakly conserved (.) amino acids are marked. Five in-frame methionine residues conserved between all three viruses at positions 57, 106, 170, 180 and 192, which may be suggestive of the generation of shorter M protein translation products, are marked in red.
